# Isolated Coarctation Repair in Neonates and Infants Through Left Thoracotomy: Short-Term Outcomes

**DOI:** 10.21470/1678-9741-2020-0554

**Published:** 2021

**Authors:** Alexandre Noboru Murakami, Ulisses Alexandre Croti, Francisco Candido Monteiro Cajueiro, Grace Arteaga, Roxann Barnes Pike, Airton Camacho Moscardini, Carlos Henrique De Marchi, Mariana Ribeiro Rodero Cardoso, Fernando Cesar Gimenes Barbosa Santos, Bruna Cury Borim

**Affiliations:** 1 Cardiology Surgery Department, Serviço de Cirurgia Cardíaca do Norte do Paraná, Universidade Estadual de Londrina (UEL), Londrina, Paraná, Brazil.; 2 Pediatric Cardiology and Cardiovascular Surgery Department, Serviço de Cardiologia e Cirurgia Cardiovascular Pediátrica de São José do Rio Preto, Hospital da Criança e Maternidade de São José do Rio Preto, Fundação Faculdade Regional de Medicina de São José do Rio Preto (FUNFARME) and Faculdade de Medicina de São José do Rio Preto (FAMERP), São José do Rio Preto, São Paulo, Brazil.; 3 Department of Pediatric and Adolescent Medicine, Division of Pediatric Critical Care Medicine, Mayo Clinic, Rochester, Minnesota, United States of America.; 4 Department of Pediatric and Adolescent Medicine, Children’s Center, Anesthesiology and Perioperative Medicine, Mayo Clinic, Rochester, Minnesota, United States of America.

**Keywords:** Aortic Coarctation, Thoracotomy, Heart Defects, Aorta, Anastomosis, Surgical, Morbidity

## Abstract

**Introduction:**

End-to-end anastomosis and extended end-to-end anastomosis are typically used as surgical approaches to coarctation of the aorta (CoAo) with access at the subclavian artery or an interposition graft. The objective of this study is to analyze the impact of surgical and anatomical characteristics and techniques on early outcomes after surgical treatment of CoAo without cardiopulmonary bypass through left thoracotomy.

**Methods:**

This is a quantitative, observational, and cross-sectional analysis of patients who underwent repair of CoAo between July 1, 2010 and December 31, 2017. Seventy-two patients were divided into three groups according to age: 34 in group A (≤ 30 days), 24 in group B (31 days to one year), and 14 in group C (≥ 1 year to 18 years).

**Results:**

Aortic arch hypoplasia was associated in 30.8% of the cases, followed by ventricular septal defect (13.2%). The preductal location was more frequent in group A (73.5%), ductal in group B (41.7%), and postductal in group C (71.4%). Long coarcted segment was predominant in groups A and C (61.8% and 71.4%, respectively) and localized in group B (58.3%). Extended end-to-end anastomosis technique was prevalent (68%), mainly in group A (91.2%). Mortality in 30 days was 1.4%.

**Conclusion:**

Most of the patients were children under one year of age, and extended end-to-end anastomosis was the most used technique, secondary to arch hypoplasia. Further, overall mortality was low in spite of moderate morbidity in the first 30 postoperative days.

**Table:** 

Abbreviations, acronyms & symbols	
CoAo	= Coarctation of the aorta
CPB	= Cardiopulmonary bypass
FAMERP	= Faculdade de Medicina de São José do Rio Preto
FUNFARME	= Fundação Faculdade Regional de Medicina de São José do Rio Preto
ICU	= Intensive care unit
IQIC	= International Quality Improvement Collaborative
MV	= Mechanical ventilation
PDA	= Persistence of ductus arteriosus
PTFE	= Polytetrafluoroethylene
SD	= Standard deviation
VAP	= Ventilator-associated pneumonia

## INTRODUCTION

Coarctation of the aorta (CoAo) can be surgically treated by median sternotomy or lateral thoracotomy, with or without cardiopulmonary bypass (CPB). The chosen surgical approach depends on multiple factors, such as the patient’s age, location of the narrowing, smallest diameter, and length of the affected site (*i.e*., discreet *vs*. long segment arch hypoplasia)^1^.

The chosen surgical technique must eliminate the obstruction, especially in children, in order to provide the most potential for tissue growth. Maneuvers with extensive resections of ductal tissue, dissections, and anastomoses with a largest possible area are essential to prevent late complications such as restenosis at the anastomosis^2^.

Left thoracotomy is usually performed without CPB. Typically, the main surgical techniques include end-to-end anastomosis and extended end-to-end anastomosis, using the subclavian artery (Waldhausen/Teles Mendonça) or an interposition graft^3,4,5^.

The study aimed to analyze surgical aspects, techniques, and early outcomes after surgical treatment of CoAo without CPB through left thoracotomy.

## METHODS

This study was a quantitative, observational, and cross-sectional analysis. It was conducted at the Serviço de Cardiologia e Cirurgia Cardiovascular Pediátrica de São José do Rio Preto, São José do Rio Preto (São Paulo, Brazil), from July 1, 2010 to December 31, 2017. Out of 1,284 patients, 93 (7.2%) underwent surgical treatment for correction of CoAo, and 72 (5.6%) of these underwent surgical treatment without CPB through left thoracotomy. The remainder were treated with CPB and sternotomy, being excluded from the study.

Patients were divided into three groups according to age: group A (≤ 30 days), 34 patients; group B (31 days to one year), 24 patients; and group C (> 1 year to 18 years), 14 patients.

Data of the International Quality Improvement Collaborative (IQIC) database (Boston Children’s Hospital at Harvard Medical School) were analyzed and collected from electronic patient records at Hospital de Base and Hospital da Criança e Maternidade de São José do Rio Preto (Fundação Faculdade Regional de Medicina de São José do Rio Preto [FUNFARME]), Faculdade de Medicina de São José do Rio Preto (FAMERP) and sent via REDCap® platform to the IQIC group.

Preoperative data included gender, prematurity, mean weight and height, and noncardiac or chromosomal abnormalities.

Intraoperative data included the diagnosis of associated diseases, section or preservation of the subclavian artery, types (discrete or long-segment) and location (preductal, ductal, or postductal), surgical techniques - including extended end-to-end anastomosis, end-to-end anastomosis, end-to-end anastomosis using the subclavian artery (Waldhausen/Teles Mendonça), and graft interposition of polytetrafluoroethylene (PTFE) tube -, and mean time of aortic clamping. Descriptions of surgical procedures were analyzed for detailed intraoperative information.

Persistence of ductus arteriosus (PDA) was not considered an associated disease, regardless of whether it was patent or occluded at the time of surgery.

Postoperative data included complications such as bacterial sepsis (presumed or confirmed by laboratory), surgical site infection (superficial, deep, or organ-to-space), and other infections (*e.g*., enterocolitis or ventilator-associated pneumonia [VAP]), total mechanical ventilation (MV) time, length of stay in intensive care unit (ICU), and mortality.

Results were presented as absolute number and percentage for the qualitative variables and mean ± standard deviation and median for the quantitative variables. For comparative analysis of qualitative variables, Fisher’s exact test was used. A *P*-value of 0.05 was considered significant.

Study protocol 3.146.205 was approved by the ethics and research committee, FUNFARME/FAMERP (CAAE number: 04379218.1.0000.5415).

There was no need to sign free and informed consent term since it only involved data collection of patients’ electronic records, without direct contact with participants.

## RESULTS

Among the preoperative data shown in Table 1, there was a slight predominance of males (56.9%). Prematurity was present in almost 10% of the patients. Surgical correction occurred on the first year of life of 58 patients in groups A and B (80.6%). Mean ± standard deviation of weight for groups A and B was 3.2±0.5 and 4.7±2 kg, respectively, confirming most children were only a few months old.

**Table 1:** Preoperative data of patients who underwent surgical treatment for coarctation of the aorta.

Preoperative data	Group A (n=34)	Group B (n=24)	Group C (n=14)	Total (n=72)
Male	19 (55.9%)	13 (54.2%)	9 (64.3%)	41 (56.9%)
Prematurity	3 (8.8%)	4 (16.7%)	0	7 (9.7%)
Weight (mean±SD) [median]	(3.2 ± 0.5) [3.3]	(4.7 ± 2) [4.5]	(23.8 ± 14.8) [18.2]	(7.7 ± 10.2) [3.7]
Height (mean±SD) [median]	(48.6 ± 3.6) [49]	(57.2 ± 10.7) [55.5]	(114 ± 28.9) [108.5]	(64.2 ± 28.6) [51]
Noncardiac anomalies	3 (8.8%)	3 (12.5%)	0	6 (8.3%)
Chromosomal abnormalities	7 (20.5%)	4 (16.7%)	1 (7.1%)	12 (16.7%)

SD=standard deviation

Group A=≤ 30 days; group B=31 days to 1 year; group C= >1 year to 18 years

Noncardiac abnormalities occurred in three patients (8.8%) in group A (Dandy Walker syndrome, cleft lip, and pyelocaliceal dilation) and three patients (12.5%) in group B, (Hirschsprung’s disease, cerebral ventricular dilation, and hypospadias). There were no associated noncardiac abnormalities in older patients (group C).

There were 12 (16.7%) chromosomal abnormalities in total, seven (20.5%) in group A, four (16.7%) in group B, and one (7.1%) in group C, suggesting the combination of chromosomal abnormalities and CoAo is operated, for the most part, up to one year of age. The most common syndromes were Turner, Down, Williams, and Pierre Robin.

Preoperative left ventricular dysfunction was present in 12.1% of the patients.

Aortic arch hypoplasia was associated in approximately one third of the patients (30.8%), followed by ventricular septal defect (13.2%), Shone’s syndrome, and atrial septal defect (both 11%). Other diagnoses were less frequent, as observed in Table 2.

**Table 2:** Main preoperative diagnosis of cardiac lesions associated to coarctation of the aorta.

Associated diagnosis*	Group A	Group B	Group C	Total
Aortic arch hypoplasia	18 (34%)	5 (18.5%)	5 (45%)	28 (30.8%)
Ventricular septal defect	9 (17%)	2 (7.4%)	1 (9%)	12 (13.2%)
Shone's syndrome	5 (9.4%)	4 (14.8%)	1 (9%)	10 (11%)
Atrial septal defect	7 (13.2%)	3 (11.1%)	0	10 (11%)
Bicuspid aortic valve	2 (3.8%)	2 (7.4%)	1 (9%)	5 (5.5%)
Ebstein's anomaly	1 (1.9%)	1 (3.7%)	0	2 (2.2%)
Subvalvar aortic stenosis	0	0	1 (9%)	1 (1.1%)
Corrected transposition of the great arteries	1 (1.9%)	0	0	1 (1.1%)
Partial atrioventricular septal defect	0	1 (3.7%)	0	1 (1.1%)
Total atrioventricular septal defect	1 (1.9%)	0	0	1 (1.1%)
Unbalanced atrioventricular septal defect	1 (1.9%)	0	0	1 (1.1%)
Aortic valve annulus hypoplasia	1 (1.9%)	0	0	1 (1.1%)
Pulmonary valvar stenosis	1 (1.9%)	0	0	1 (1.1%)
Coronary-cavitary fistula	1 (1.9%)	0	0	1 (1.1%)
Parachute mitral valve	0	1 (3.7%)	0	1 (1.1%)
Mitral stenosis	0	1 (3.7%)	0	1 (1.1%)
Aberrant right subclavian	1 (1.9%)	0	0	1 (1.1%)
Total	53 (100%)	27 (100%)	11 (100%)	91 (100%)

* Persistence of ductus arteriosus was not considered an associated disease, regardless of whether it was patent or occluded at the time of surgery.

Shone’s syndrome diagnosis included at least three of the following anatomic findings: mitral stenosis, other left-sided obstructive lesions such as supramitral ring, valvular aortic stenosis, subaortic stenosis, and CoAo^6^.

In the intraoperative period, the most common location and types of CoAo observed during the surgical procedure are shown in Table 3. Preductal location was more frequent in group A (73.5% of neonates), ductal location in group B (41.7%), and postductal in group C (71.4%). Long-segment narrowing predominated in groups A and C (61.8% and 71.4%, respectively) and discrete in group B (58.3%).

**Table 3:** Location and type of coarctation of the aorta during operation.

Group	Location			Type	
	Preductal	Ductal	Postductal	Discrete	Long-segment
A (n=34)	25 (73.5%)	6 (17.6%)	3 (8.8%)	13 (38.2%)	21 (61.8%)
B (n=24)	5 (20.8%)	10 (41.7%)	9 (37.5%)	14 (58.3%)	10 (41.7%)
C (n=14)	1 (7.1%)	3 (21.4%)	10 (71.4%)	4 (28.6%)	10 (71.4%)

The surgical techniques used for correction of CoAo are described in Table 4.

**Table 4:** Surgical techniques for coarctation of the aorta repair.

Procedures	Group A (n=34)	Group B (n=24)	Group C (n=14)	Total
Extended end-to-end anastomosis	31 (91.2%)	15 (62.5%)	3 (21.4%)	49 (68%)
End-to-end anastomosis	2 (5.9%)	9 (37.5%)	7 (50%)	18 (25%)
Graft interposition of PTFE tube	0	0	2 (14.3%)	2 (2.8%)
End-to-end anastomosis using the subclavian artery (Waldhausen)	0	0	2 (14.3%)	2 (2.8%)
End-to-end anastomosis using the subclavian artery (Teles Mendonça)	1 (2.9%)	0	0	1 (1.4%)
Total	34 (100%)	24 (100%)	14 (100%)	72 (100%)

n=number of patients; PTFE=polytetrafluoroethylene

The most frequent surgical technique in all groups was the extended end-to-end anastomosis (68%). It was more prevalent in group A (91.2%). For group C, half of the operated patients underwent end-to-end anastomosis, which was the second most commonly used technique in all groups.

End-to-end anastomosis using the subclavian artery, the Waldhausen technique, was performed only in group C, and Teles Mendonça technique only in group A. We used interposition of PTFE tube grafts only in the older patients from group C (14.3%).

The subclavian artery was excluded in eight patients (23.5%) in group A and two patients (35.7%) in group C.

The mean aortic cross-clamping time for each group was expressed in minutes and is shown in Table 5.

**Table 5:** Mean aortic clamping time for coarctation of the aorta repair.

Group A (n=34)	17 minutes
Group B (n=24)	14 minutes
Group C (n=14)	18 minutes
Total (n=72)	16 minutes

n=number of patients

**Fig. 1:**
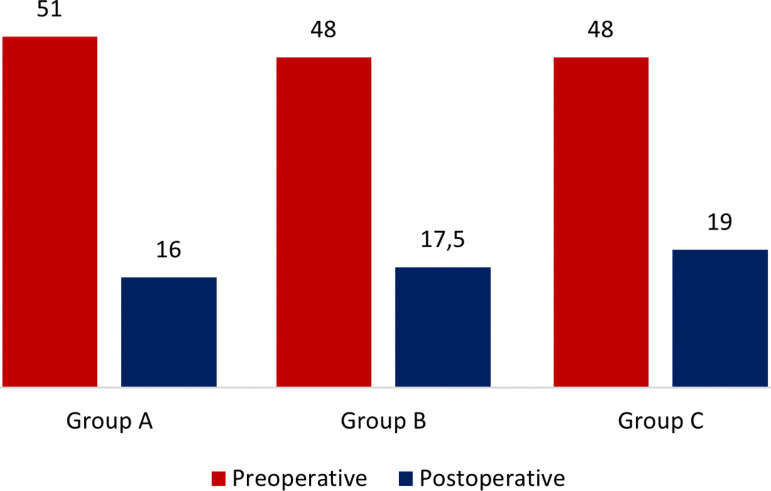
Median pre and postoperative aortic arch gradients (in mmHg) for each group.

The median pre and postoperative aortic arch gradients for each group is illustrated in Figure 1.

The most relevant postoperative information demonstrating early outcomes within 30 days is shown in Table 6.

**Table 6:** Postoperative and immediate mortality data post coarctation of the aorta repair.

Postoperative data	Group A (n=34)	Group B (n=24)	Group C (n=14)	Total (n=72)
MV time, hours (mean±SD) [median]	(140.1±256.1) [55.1]	(66.2±120.3) [21.1]	(12.5±30.5) [3.7]	(89.2±192.5) [26.1]
Bacterial sepsis	7 (20.6%)	4 (16.7%)	0	11 (15.3%)
Surgical site infection	4 (11.8%)	1 (4.2%)	1 (7.1%)	6 (8.3%)
Other infections	2 (5.9%)	5 (20.8%)	1 (7.1%)	8 (11.1%)
ICU time, hours (mean±SD) [median]	(330.3±341.4) [194.1]	(339.9±284.8) [156.8]	(142.5±129.6) [88.4]	(261.8±296.3) [167.1]
30-day mortality	0	1 (4.2%)	0	1 (1.4%)

ICU=intensive care unit; MV=mechanical ventilation; SD=standard deviation

The mean duration of MV and ICU times was higher in group A, including neonates. It was approximately six days for MV and 14 days for ICU.

Regarding infection, 15.3% were presumed or laboratory confirmed bacterial sepsis, mostly present in group A (20.6%).

After data analysis of bacterial sepsis present in the three groups, we observed that of the seven patients in group A, only two had laboratory confirmed bacterial sepsis (*Klebsiella pneumoniae* and *Acinetobacter baumannii*). In group B, four patients (16.7%) were diagnosed with presumed bacterial sepsis, though all cultures were negative. None of the patients from group C exhibited any clinical signs or symptoms concerning for bacteremia.

Six patients (8.3%) were diagnosed with surgical site infection, four (11.8%) in group A and one in groups B and C (4.2% and 7.1%, respectively).

Other infections included ventilator-associated pneumonia and enterocolitis in group A (5.9%), enterocolitis, tracheobronchitis, pneumonia, and two patients with VAP in group B (20.8%), and a case of pneumonia (not ventilation acquired) in group C (7.1%).

There was no statistically significant difference between groups with regard to bacterial sepsis or surgical site infection, as shown in Table 7.

**Table 7:** Comparative analysis of variables related to infection among groups of patients submitted to surgical repair of coarctation of the aorta.

Variable	*P*-value
**Bacterial sepsis**	
Group A × Group B	0.731
Group A × Group C	0.073
Group B × Group C	0.144
**Surgical site infection**	
Group A × Group B	0.364
Group A × Group C	0.704
Group B × Group C	0.7368
**Other infections**	
Group A × Group B	0.112
Group A × Group C	0.854
Group B × Group C	0.313

Group A=34 patients; group B=24 patients; group C=14 patients

Mortality at 30 days occurred in only one patient in group B (4.2%) due to infection. There were no deaths in groups A or C; thus, the mortality for all groups combined (72 patients) was 1.4%.

## DISCUSSION

CoAo is often associated with chromosomal and other intracardiac abnormalities, and in a Brazilian cohort, 50% of operated patients presented, in descending order, patent ductus arteriosus, ventricular septal defect, and bicuspid aortic valve^7^. Aortic arch hypoplasia is usually the most commonly associated (81%) and rarely found as a single diagnosis^8^. Similar results were found in the present study.

It is important for patients to have a detailed evaluation of their aortic arch and to rule out any associated cardiac anomalies, given the influence that information has on surgical planning. Surgeons rely on accurate assessment of cardiac and arch anatomy for making decisions regarding incision location, surgical approach (especially if there are additional lesions that need to be addressed), and CPB cannulation^9^.

The classification of CoAo location is based on the location of the ductus arteriosus. Preductal CoAo is more frequent in fetal and neonatal patients, compatible with this study’s results (73.5%)^10^.

Surgical outcomes continue to improve. However, associated CoAo and aortic arch hypoplasia treatment is controversial. The Texas Children’s Hospital group favors median sternotomy for proximal hypoplasia and lateral thoracotomy is limited to transverse arch hypoplasia and or high surgical risk patients for intervention with CPB^1^.

Left thoracotomy and end-to-end anastomosis is the option of choice for optimal survival, which is > 90% in isolated CoAo. When associated with other complex abnormalities, the mortality rate increases to 20%^9^.

A study published in 2019 states that extended end-to-end anastomosis is the best surgical approach for infants and children with simple CoAo. Even with transverse arch hypoplasia, left thoracotomy repair has low mortality (0%), reintervention rates (2%), and low incidence of hypertension (18% in median of 5.4 years). Median sternotomy should be considered for patients with a distal transverse arch diameter with a *z*-score < 2.8 and proximal transverse arch diameter *z*-score < 4.1^2^.

In our study, most patients underwent extended end-to-end anastomosis; notably, in neonates (91.2%) and < 1 year (62.5%) patients, given the association of aortic arch hypoplasia (need for oblique arch enlargement) in 30.8% of all patients.

Similar data are found in the Society of Thoracic Surgeons (or STS) Congenital Heart Surgery Database, in a cohort of 5,025 patients from 95 American centers who underwent CoAo surgical correction from 2006 to 2010. The most common techniques for repair of CoAo and aortic arch hypoplasia were extended end-to-end anastomosis (56%) and end-to-end anastomosis (33%), with a total mortality of 2.4%^11^.

Mery et al.^1^ analyzed 343 patients, age up to 18 years, 42% of whom were newborns and 36% < 1 year old submitted to surgical correction of CoAo through left thoracotomy. Extended end-to-end anastomosis was performed in 85% of the total, followed by end-to-end anastomosis (13%), subclavian flap (2%), and interposition of tubular graft (0.6%). The conclusion was that repair through left thoracotomy is associated with lower morbidity, mortality, and reintervention rates at a mean follow-up of six years.

In our service, regardless of age, we recommend surgical correction of CoAo as soon as the diagnosis is made. A study conducted in the Netherlands, between 2010 and 2016, analyzed 213 neonates and 85 infants < 6 months of age and had similar data to those found in the present study. The median weights were 3.4 kg and 4.4 kg. Postoperative ICU admission time was 2.72 days and length of hospital stay of 5.81 days. Newborns presented greater ICU and longer hospital stay when compared to infants. They concluded that coarctation repair could be can be safely performed in neonates and infants^12^.

Mohammed et al.^13^, compared early extubation strategy in the operating room (fast-track) and subsequent weaning in the ICU in patients submitted to surgical correction of CoAo through left thoracotomy. The immediate extubation protocol in the operating room did not present advantages. The authors reported more frequent use of vasodilators for blood pressure and higher doses of opioids causing respiratory depression and reinterventions, higher rate of reoperation due to bleeding associated to uncontrolled hypertension, and no decrease in the average length of ICU stay.

Compared to patients with isolated CoAo, those with CoAo in the setting of other cardiac anomalies have a higher incidence of complications (*i.e.*, perioperative acidosis, cardiac arrest, chylothorax) and more frequently require prolonged MV. Furthermore, patients with CoAo and additional cardiac anomalies have residual lesions that necessitate reoperation^11^.

In a study published in 2009 with 201 children, 78% of the CoAo repairs were extended end-to-end anastomosis technique with left lateral thoracic pathway and without CPB, similar to this study (total of 68%). In this population, mean aortic cross-clamping time was 18 ± 4 minutes (ranging from 10 to 41 minutes), most common complications were sepsis (4%), recurrent laryngeal nerve palsy (3%), chylothorax (3%), and pulmonary hypertension (1%), and mean hospital stay was seven days^13^.

Hospital mortality in the last 20 years has been low (2 to 10%) in neonates who undergo surgical correction with or without PDA. More recently, some specialized centers have reported mortality rates as low as 0-2%. When CoAo repair is performed on older infants, children, adolescents, or young adults, early mortality rate is around 1%^14^. Our 30-day mortality for the entire cohort, overall, was 1.4%.

## CONCLUSION

Most of the patients were children under one year of age, and extended end-to-end anastomosis was the most used technique, secondary to arch hypoplasia. Further, overall mortality was low in spite of moderate morbidity in the first 30 postoperative days.

**Table:** 

Authors' roles & responsibilities			
ANM	Substantial contributions to the conception or design of the work; or the acquisition, analysis, or interpretation of data for the work; drafting the work or revising it critically for important intellectual content; agreement to be accountable for all aspects of the work in ensuring that questions related to the accuracy or integrity of any part of the work are appropriately investigated and resolved; final approval of the version to be published	ACM	Substantial contributions to the conception or design of the work; or the acquisition, analysis, or interpretation of data for the work; drafting the work or revising it critically for important intellectual content; agreement to be accountable for all aspects of the work in ensuring that questions related to the accuracy or integrity of any part of the work are appropriately investigated and resolved; final approval of the version to be published
UAC	Substantial contributions to the conception or design of the work; or the acquisition, analysis, or interpretation of data for the work; drafting the work or revising it critically for important intellectual content; agreement to be accountable for all aspects of the work in ensuring that questions related to the accuracy or integrity of any part of the work are appropriately investigated and resolved; final approval of the version to be published	CHM	Substantial contributions to the conception or design of the work; or the acquisition, analysis, or interpretation of data for the work; drafting the work or revising it critically for important intellectual content; agreement to be accountable for all aspects of the work in ensuring that questions related to the accuracy or integrity of any part of the work are appropriately investigated and resolved; final approval of the version to be published
FCMC	Substantial contributions to the conception or design of the work; or the acquisition, analysis, or interpretation of data for the work; drafting the work or revising it critically for important intellectual content; agreement to be accountable for all aspects of the work in ensuring that questions related to the accuracy or integrity of any part of the work are appropriately investigated and resolved; final approval of the version to be published	MRRC	Substantial contributions to the conception or design of the work; or the acquisition, analysis, or interpretation of data for the work; drafting the work or revising it critically for important intellectual content; agreement to be accountable for all aspects of the work in ensuring that questions related to the accuracy or integrity of any part of the work are appropriately investigated and resolved; final approval of the version to be published
GA	Substantial contributions to the conception or design of the work; or the acquisition, analysis, or interpretation of data for the work; drafting the work or revising it critically for important intellectual content; agreement to be accountable for all aspects of the work in ensuring that questions related to the accuracy or integrity of any part of the work are appropriately investigated and resolved; final approval of the version to be published	FCGBS	Substantial contributions to the conception or design of the work; or the acquisition, analysis, or interpretation of data for the work; drafting the work or revising it critically for important intellectual content; agreement to be accountable for all aspects of the work in ensuring that questions related to the accuracy or integrity of any part of the work are appropriately investigated and resolved; final approval of the version to be published
RBP	Substantial contributions to the conception or design of the work; or the acquisition, analysis, or interpretation of data for the work; drafting the work or revising it critically for important intellectual content; agreement to be accountable for all aspects of the work in ensuring that questions related to the accuracy or integrity of any part of the work are appropriately investigated and resolved; final approval of the version to be published	BCB	Substantial contributions to the conception or design of the work; or the acquisition, analysis, or interpretation of data for the work; drafting the work or revising it critically for important intellectual content; agreement to be accountable for all aspects of the work in ensuring that questions related to the accuracy or integrity of any part of the work are appropriately investigated and resolved; final approval of the version to be published
